# *CYP2D6* Polymorphisms and the Safety and Gametocytocidal Activity of Single-Dose Primaquine for Plasmodium falciparum

**DOI:** 10.1128/AAC.00538-19

**Published:** 2019-09-23

**Authors:** Helmi Pett, John Bradley, Joseph Okebe, Alassane Dicko, Alfred B. Tiono, Bronner P. Gonçalves, Will Stone, Ingrid Chen, Kjerstin Lanke, Mikko Neuvonen, Anna-Liina Mustaniemi, Alice C. Eziefula, Roly Gosling, Umberto D’Alessandro, Chris Drakeley, Mikko Niemi, Teun Bousema

**Affiliations:** a Radboud Institute for Health Sciences, Radboud University Medical Center, Nijmegen, the Netherlands; b Department of Clinical Pharmacology, University of Helsinki and Helsinki University Hospital, Helsinki, Finland; c MRC Tropical Epidemiology Group, London School of Hygiene and Tropical Medicine, London, United Kingdom; d Medical Research Council Unit the Gambia at the London School of Hygiene and Tropical Medicine, Banjul, the Gambia; e Malaria Research and Training Centre, Faculty of Pharmacy and Faculty of Medicine and Dentistry, University of Science, Techniques and Technologies of Bamako, Bamako, Mali; f Public Health Department, Centre National de Recherche et de Formation sur le Paludisme, Ouagadougou, Burkina Faso; g Department of Immunology and Infection, London School of Hygiene and Tropical Medicine, London, United Kingdom; h Malaria Elimination Initiative, Global Health Group, University of California, San Francisco, San Francisco, California, USA; i Individualized Drug Therapy Research Program, Faculty of Medicine, University of Helsinki, Helsinki, Finland; j Department of Forensic Medicine, University of Helsinki, Helsinki, Finland; k Brighton and Sussex Centre for Global Health Research, Brighton and Sussex Medical School, University of Sussex, Brighton, United Kingdom

**Keywords:** cytochrome P450, drug metabolism, elimination, gametocyte, genetic polymorphisms, malaria, metabolite, primaquine, safety, transmission

## Abstract

Single-dose primaquine (PQ) clears mature gametocytes and reduces the transmission of Plasmodium falciparum after artemisinin combination therapy. Genetic variation in *CYP2D6*, the gene producing the drug-metabolizing enzyme cytochrome P450 2D6 (CYP2D6), influences plasma concentrations of PQ and its metabolites and is associated with PQ treatment failure in Plasmodium vivax malaria. Using blood and saliva samples of varying quantity and quality from 8 clinical trials across Africa (*n* = 1,076), we were able to genotype *CYP2D6* for 774 samples (72%). We determined whether genetic variation in *CYP2D6* has implications for PQ efficacy in individuals with gametocytes at the time of PQ administration (*n* = 554) and for safety in glucose-6-phosphate dehydrogenase (G6PD)-deficient individuals treated with PQ (*n* = 110). Individuals with a genetically inferred CYP2D6 poor/intermediate metabolizer status had a higher gametocyte prevalence on day 7 or 10 after PQ than those with an extensive/ultrarapid CYP2D6 metabolizer status (odds ratio [OR] = 1.79 [95% confidence interval {CI}, 1.10, 2.90]; *P* = 0.018). The mean minimum hemoglobin concentrations during follow-up for G6PD-deficient individuals were 11.8 g/dl for CYP2D6 extensive/ultrarapid metabolizers and 12.1 g/dl for CYP2D6 poor/intermediate metabolizers (*P* = 0. 803). CYP2D6 genetically inferred metabolizer status was also not associated with anemia following PQ treatment (*P* = 0.331). We conclude that CYP2D6 poor/intermediate metabolizer status may be associated with prolonged gametocyte carriage after treatment with single-low-dose PQ but not with treatment safety.

## Abstract (precis)

Single-dose primaquine (PQ) clears mature gametocytes and reduces the transmission of Plasmodium falciparum after artemisinin combination therapy. Genetic variation in *CYP2D6*, the gene producing the drug-metabolizing enzyme cytochrome P450 2D6 (CYP2D6), influences plasma concentrations of PQ and its metabolites and is associated with PQ treatment failure in Plasmodium vivax malaria.

## INTRODUCTION

With recent successes in malaria control and the move toward Plasmodium falciparum elimination, there is an increasing interest in transmission-reducing strategies. One of the tools available is single-low-dose (0.25 mg/kg of body weight) primaquine (PQ) added to artemisinin combination therapy (ACT). PQ is a drug in the class of 8-aminoquinolines that has been on the market for more than 70 years. In recent years, the addition of PQ to ACTs has received considerable interest because of its ability to rapidly clear mature P. falciparum gametocytes and reduce the infectious period compared to ACT alone (1–6).

Cytochrome P450 2D6 (CYP2D6) is a human enzyme involved in the metabolization of 20 to 25% of all prescribed medicines (7–10). Hundreds of different *CYP2D6* alleles have been discovered, some of which influence the activity of the produced enzyme (11). Bennett and colleagues first associated genetic *CYP2D6* variation with relapses of Plasmodium vivax malaria after PQ treatment (12). More recently, genetic *CYP2D6* variation was found to be strongly associated with an increased risk of relapses among Indonesian patients with clinical P. vivax malaria (7). There is also evidence in mice that enzymes in the CYP2D family produce the active metabolite of PQ against Plasmodium berghei liver stages (13), but metabolic activation of PQ may not be necessary to eradicate blood stages (14).

The implications of genetic *CYP2D6* variation for the use of PQ in P. falciparum infections have never been explored. One of the factors that has hindered the widespread adoption of PQ for P. falciparum transmission reduction is its safety profile, notably in individuals with genetic deficiencies in glucose-6-phosphate dehydrogenase (G6PD) production (12, 15–17). G6PD is an enzyme involved in the pentose phosphate pathway in human red blood cells (18), and G6PD deficiency (G6PDd) is associated with hemolysis following treatment with PQ. Despite safety concerns related to the hemolytic activity of PQ in individuals with G6PDd, a single low dose of PQ is considered safe in individuals with the most common African G6PDd variant (G6PDd A variant) (19–21). Since genetic variation in *CYP2D6* influences the pharmacokinetics of single-low-dose PQ in humans (22), this variation may have implications for PQ efficacy or safety at doses targeting P. falciparum gametocytes.

Here, we determine the impact of genetically inferred CYP2D6 metabolizer status on the gametocytocidal and hemolytic effect of single-dose PQ in 8 clinical trials conducted across Africa.

## RESULTS

**FIG 1:**
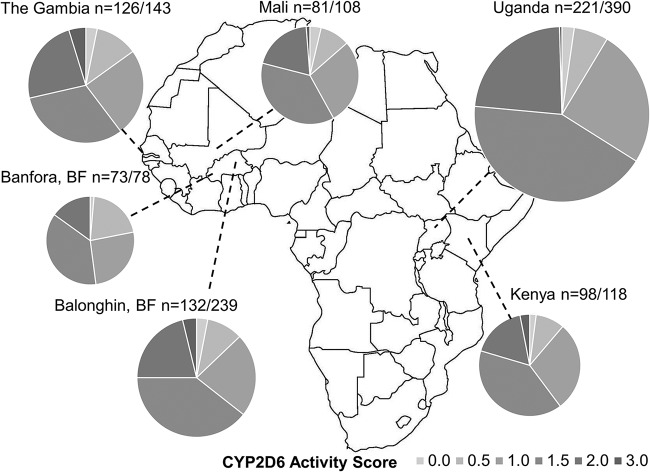
Genotypically inferred CYP2D6 AS for six African populations. Only samples for which an exact AS was inferred are included (*n*/*N*, where *n* is the number of individuals for whom an AS was determined and *N* is the number of samples for which genotyping was attempted). It was not possible to infer ASs for all individuals with a determined genotype due to not knowing which haplotype is duplicated (see Data Set S1 in the supplemental material). In some cases, it was possible to determine an AS range (Data Set S2) but not an exact AS. For Mali and the Gambia, results from efficacy and safety studies were combined; in Burkina Faso (BF), the efficacy and safety studies were carried out in two distinct populations in different areas, and therefore, the AS results are presented separately for the Balonghin and Banfora populations.

*CYP2D6* genotyping with the OpenArray technology used here requires high-quality DNA, ideally 50 ng/μl, a condition that was not always met. *CYP2D6* genotyping was thus successful in 72% (774/1,076) of all samples; success varied considerably between sample types, with good success rates for saliva samples (≥98%) and large-volume blood samples (≥0.5 ml blood) (success rate of ≥87%) but low success rates for different sample types (1 to 68%) (Table 1; see also Data Set S1 in the supplemental material). As a result of differences in sample collection methods between sites, genotyping was successful for ≤58% of samples from Uganda and Balonghin, Burkina Faso, but for ≥80% of samples for other sites (Table 1 and Data Set S1). Inference of the CYP2D6 activity score (AS) from genotypes was successful in 68% (731/1,076) of samples and is presented for the different sites in Fig. 1. The CYP2D6 AS inference allowed classification of sample donors as poor metabolizer (PM) (activity score of 0), intermediate metabolizer (IM) (activity score of 0.5 to 1.0), extensive metabolizer (EM) (activity score of 1.5 to 2.0), or ultrarapid metabolizer (UM) (activity score of >2.0). For other samples, a range of ASs could be inferred that allowed classification into EM/UM classes (AS ≥ 1.5; *n* = 137) (Data Set S2). CYP2D6 PM status was inferred for a minority of individuals (2.6%; 19/731); CYP2D6 IM status was inferred for 38.2% of individuals (279/731).

**TABLE 1:** Trial details and samples available^*a*^

Country, study type (reference)	Falciparum malaria status	G6PDd status	ACT	PQ timing (day)	PQ dose(s) (mg/kg)	Days of gametocyte measurement	Days of hemoglobin measurement	CYP2D6		No. of samples included	
								Sample type(s) (no. of samples)	% genotyping success (no. of samples)	Efficacy	Safety
Uganda, efficacy (2)	Uncomplicated malaria	Normal by fluorescent spot test	AL	2	0.75, 0.4, 0.1	0, 2, 3, 7, 10, 14	0, 1, 2, 3, 7, 10, 14, 21	50 μl EDTA blood in L6 (345), filter paper (45)	58 for blood in L6 (226), filter paper (2)	138	11
Burkina Faso (Balonghin), efficacy (3)	Asymptomatic infection	Normal by rapid diagnostic test	AL	2	0.4, 0.25	0, 7	0, 1, 2, 3, 7, 10, 14	100 μl EDTA blood in RNAprotect, (100), 0.5–1 ml EDTA blood (112), Oragene saliva samples (27)	57 for blood in RNAprotect (1), EDTA blood (109), Oragene saliva samples (27)	182	8
Burkina Faso (Banfora), safety (19)	Asymptomatic infection	Deficient by fluorescent spot test (and controls)	AL	0	0.4, 0.25	0, 3, 7	0, 1, 2, 3, 4, 5, 7, 10, 14, 28	0.5–1 ml EDTA blood (78)	97 (76)	0	43
Kenya, efficacy (5)	Asymptomatic gametocyte carrier	Regardless of G6PD status	DP	2	0.25	0, 2, 3, 7, 14	0, 2, 3, 7, 14	0.5–1 ml EDTA blood (118)	87 (103)	99	7
Mali, efficacy (1)	Asymptomatic gametocyte carrier	Normal by colorimetric quantification	DP	0	0.5, 0.25, 0.125, 0.0625	0, 2, 3, 7, 14, 28	0, 1, 2, 3, 7, 14, 28	50 μl EDTA blood in L6 (47), blood pellets (33)	80 for blood in L6 (32), blood pellets (32)	56	4
Mali, safety (20)	Parasite free (by microscopy)	Normal controls and deficient by rapid diagnostic test	None	0	0.5, 0.45, 0.4	None	0, 1, 2, 3, 4, 5, 6, 7, 8, 9, 10, 14, 28	0.5–1 ml EDTA blood (28)	93 (26)	0	18
The Gambia, efficacy (4)	Asymptomatic infection	Normal by fluorescent spot test	DP	2	0.75, 0.4, 0.2	0, 3, 7, 10, 14	0, 1, 2, 3, 7, 10, 14, 21, 28, 35, 42	Oragene saliva samples (85)	99 (84)	69	0
The Gambia, safety (19)	Regardless of infection status	Deficient by fluorescent spot test (and controls)	DP	0	0.4, 0.25	0, 3, 7	0, 1, 2, 3, 4, 5, 7, 10, 14, 28	0.5–1 ml EDTA blood (58)	97 (56)	0	19

a AL, artemether-lumefantrine; DP, dihydroartemisinin-piperaquine.

A total of 544 participants from 5 studies who had gametocytes by molecular methods on the day of initiation of treatment, completed treatment, and had complete outcome measures were included in the efficacy analysis. The prevalence of CYP2D6 PM/IM status among these individuals was 31.4% (171/544) overall and ranged from 26% to 41% by study. Compared to ACT alone, PQ was effective in reducing gametocyte prevalence on day 7 or 10 in both CYP2D6 EM/UM (odds ratio [OR] = 0.20 [95% confidence interval {CI}, 0.11, 0.36]; *P* < 0.001) and CYP2D6 PM/IM (OR = 0.15 [95% CI, 0.05, 0.44]; *P* = 0.001) individuals. Individuals with CYP2D6 PM/IM status had a higher gametocyte prevalence at day 7 or 10 after PQ treatment than those with CYP2D6 EM/UM status (Table 2), after adjusting for PQ dose, country, and baseline gametocyte density (OR = 1.79 [95% CI, 1.10 to 2.90]; *P* = 0.018).

**TABLE 2:** Effect of CYP2D6 metabolizer status and covariates on gametocyte prevalence at day 7 or 10 among individuals receiving primaquine

Covariate	No. of individuals with gametocytes on day 7 or 10/total no. of individuals (%)	OR (95% CI)	Adjusted OR^*a*^ (95% CI)
CYP2D6 status		*P* = 0.028	*P* = 0.018
EM/UM	80/289 (28)	1	1
PM/IM	51/133 (38)	1.62 (1.05–2.50)	1.79 (1.10–2.90)

Baseline gametocyte density/ml		1.002 (1.000–1.003) (*P* = 0.005)	1.002 (1.001–1.003) (*P* = 0.004)

Baseline asexual parasite density/ml		0.998 (0.994, 1.002) (*P* = 0.380)	0.994 (0.982, 0.999) (*P* = 0.025)

PQ dose (mg/kg)		*P* < 0.001	*P* < 0.001
0.25	89/228 (39)	1	1
0.5	30/153 (20)	0.38 (0.24–0.62)	0.32 (0.18–0.56)
0.75	12/41 (29)	0.65 (0.31–1.33)	0.34 (0.14–0.82)

Country		*P* < 0.001	*P* < 0.001
Burkina Faso	26/166 (16)	1	1
Kenya	20/50 (40)	3.59 (1.78–7.26)	2.24 (1.07–4.74)
Mali	21/43 (49)	5.14 (2.48–10.66)	6.05 (2.76–13.25)
Gambia	24/61 (39)	3.49 (1.80–6.78)	3.27 (1.62–6.59)
Uganda	40/102 (39)	3.47 (1.95–6.19)	4.19 (2.03–8.64)

a Adjusted for all other factors in the table.

For the safety analysis, PQ was administered to 110 G6PDd individuals in 7 different studies. Among these PQ-treated G6PDd individuals, 56% (62/110) were EM/UM and possibly at risk of more severe hemolysis due to increased availability of the active metabolite(s) of PQ. The pretreatment mean hemoglobin (Hb) concentrations were 13.3 g/dl in the CYP2D6 EM/UM G6PDd individuals and 13.4 g/dl in the CYP2D6 PM/IM G6PDd individuals (*P* = 0.803). The mean minimum Hb concentrations during 10 to 28 days of follow-up were 11.8 g/dl for CYP2D6 EM/UM G6PDd individuals and 12.1 g/dl for CYP2D6 PM/IM G6PDd individuals. This difference, adjusted for baseline Hb concentration, country, and primaquine dose, was 0.05 g/dl (95% CI, −0.34, 0.44) (*P* = 0.803) and not statistically significant. One hundred individuals had Hb measurement on day 7 posttreatment: the mean Hb concentrations on day 7 were 12.5 g/dl for CYP2D6 EM/UM G6PDd individuals and 12.8 g/dl for CYP2D6 PM/IM G6PDd individuals (adjusted difference, 0.25 g/dl [95% CI, −0.24, 0.74]; *P* = 0.314). Twenty-four percent (15/62) of CYP2D6 EM/UM G6PDd individuals experienced moderate anemia, compared to 23% (11/48) of CYP2D6 PM/IM G6PDd individuals (adjusted odds ratio, 2.11 [95% CI, 0.46, 9.72]; *P* = 0.334). Only one G6PDd individual from Burkina Faso had severe anemia after PQ treatment (Hb concentration of 7 g/dl at day 10); this individual was CYP2D6 EM/UM, had a baseline Hb concentration of 12.5 g/dl, and recovered completely by day 14 (Hb concentration of 11.9 g/dl).

Although the *CYP2D6* genotyping success was low for some sample sets, genotyping success was not associated with persisting gametocytes on day 7 or 10 (OR = 0.95 [95% CI, 0.65 to 1.38]; *P* = 0.771) or Hb (difference of −0.83 [95% CI, −1.91, 0.25]; *P* = 0.129) in models adjusted for country, PQ dose, and baseline gametocyte density. We thus found no evidence for selection bias in our efficacy and safety outcome assessments due to variation in *CYP2D6* genotyping success.

## DISCUSSION

In the present study, we utilized samples from clinical trials across Africa to explore the effect of genetically inferred CYP2D6 metabolizer status on PQ efficacy and safety. Compared to ACT alone, the addition of single-dose PQ resulted in a marked reduction in gametocyte carriage across populations with different CYP2D6 metabolizer statuses. Nevertheless, CYP2D6 PM/IM individuals were more likely to have persisting gametocytes until day 7 or 10 following initiation of treatment with ACT-PQ.

While the transmission-blocking effect of PQ may precede the gametocyte-clearing effect and gametocytes persisting after PQ may not result in onward transmission to mosquitoes (1, 6, 23), the results of the present study suggest that the efficacy of low-dose PQ may be affected by CYP2D6 metabolizer status. We previously demonstrated that PQ pharmacokinetics are influenced by genetically inferred CYP2D6 metabolizer status (22), suggesting that lower concentrations of the PQ active metabolites may occur in CYP2D6 PM/IM individuals. While CYP2D6 metabolizer status and concentrations of active PQ metabolites have direct implications for P. vivax-infected patients by affecting cure rates (12), the effect on P. falciparum-infected patients is indirect, potentially increasing the number of secondary cases arising from a PQ-treated gametocyte carrier.

We observed no effect of CYP2D6 metabolizer status on Hb concentrations after PQ treatment of G6PDd individuals. We hypothesized that G6PDd individuals with CYP2D6 PM/IM status would be relatively protected from hemolysis, but this was not observed. While we combined data from safety studies to maximize the number of observations in G6PDd individuals, it is possible that our study population size was insufficient to detect subtle effects on hemolysis. Interstudy variation may also have obscured effects of CYP2D6 status, although study site was incorporated into our multivariate regression models.

There are several limitations to this study. We worked with available samples from several clinical trials, not specifically collecting material for extensive human genotyping. The variable quality and quantity of samples affected our genotyping success rate but are unlikely to have affected the validity of our comparisons between populations with successful genotyping results. Similarly, the present study did not allow us to detect possible differences in effects between ACTs. CYP2D6 activity and PQ metabolism may be influenced differently by dihydroartemisinin-piperaquine (DP) (24) and artemether-lumefantrine (AL) (25). While we combined findings from trials with different ACTs, this is unlikely to have affected the validity of our findings, and we adjusted for study effects. Another limitation is that we inferred CYP2D6 metabolizer status from the *CYP2D6* genotype. There has been a series of publications describing situations where the commercially available TaqMan assays, also used here in the OpenArray format, have not worked as expected and have been redesigned (26–29). Most significantly, one assay variant detecting CYP single nucleotide polymorphisms (SNPs) (*15 allele; C_32407245_40) suffers from interference from the sequence of the pseudogene *CYP2D7* to the extent that these results were not included in the analysis (27). Some additional assays have been replaced with new and improved ones during the course of this study (*7 assay [C_32388575_30 with C_32388575_A0], *8 assay [C_30634117C_20 with C_30634117C_K0], and *14 assay [C_30634117D_30 with C_30634117D_M0]) (30). In addition, a copy number variation (CNV) assay targeting intron 2 (Hs04083572_cn) may not always give the correct result due to intronic polymorphisms, and CNV assays in general work only with high sample quality (and not after product preamplification). These challenges in genetic analysis underline the complexity of the locus and the need for more sequencing of *CYP2D6*. Especially in African populations for which pharmacogenetic data are lacking, additional data are needed (31). Such future studies may purposefully collect select samples for human genotyping. In our studies, 0.5 to 1 ml blood collected in ethylenediaminetetraacetic acid (EDTA)-coated tubes or Oragene saliva samples resulted in high genotyping success rates (Table 1). Another option is to perform CYP2D6 phenotyping experiments, where a probe substrate to assess CYP2D6 activity is used. Although substrate specificity may complicate extrapolation of data from such assays to PQ metabolism, an unquestionable advantage of phenotyping is that it would take into consideration environmental factors influencing CYP2D6 activity. These include, but are not limited to, comorbidities, concomitant medication, and food intake (32, 33).

Despite limitations, including the modest number of observations from individuals with the genetically inferred CYP2D6 PM phenotype, we present evidence that CYP2D6 PM/IM status is associated with prolonged gametocyte carriage after treatment. It is currently unclear whether this has implications for the transmission-blocking effects of PQ at the population level in malaria elimination settings. A clinically meaningful effect of genetically inferred CYP2D6 metabolizer status on PQ-induced hemolysis in G6PDd individuals is unlikely.

## MATERIALS AND METHODS

### Study samples.

Samples from 8 published clinical trials were used for separate analyses on the impact of genetically inferred CYP2D6 metabolizer status on PQ safety and efficacy. For analyses of the impact of CYP2D6 inferred metabolizer status on PQ efficacy, we included samples from 5 PQ efficacy studies. Gametocyte detection was performed following treatment with a single dose of 0.1 to 0.75 mg/kg PQ in combination with either artemether-lumefantrine (AL) (Coartem as a standard 6-dose regimen over 3 days; Novartis Pharma, Switzerland) in Burkina Faso (3) and Uganda (2) or dihydroartemisinin-piperaquine (DP) (Eurartesim as a standard 3-day regimen; Sigma-Tau, Italy) in Mali (1), the Gambia (4), and Kenya (5). Analyses on the impact of CYP2D6 inferred metabolizer status on hemolysis were restricted to G6PD-deficient (G6PDd) individuals; we included two additional studies that specifically assessed PQ safety in G6PD-deficient individuals in Mali (20) and the Gambia (19), using 0.25 to 0.5 mg/kg PQ in combination with DP. In all studies, hemoglobin (Hb) concentrations in grams per deciliter were measured by a self-calibrating HemoCue photometer (Ängelholm, Sweden). Study details are summarized in Table 1.

### Extraction of nucleic acids.

An automated MagNA Pure LC 2.0 instrument (Roche, Switzerland) was used for extraction of total nucleic acid (NA) or DNA. For the samples from Uganda as well as the parasitology samples from Mali, a MagNA Pure LC high-performance total nucleic acid isolation kit was used. For samples from Burkina Faso, Kenya, and the first season of the trial in the Gambia (both full blood in EDTA and saliva samples), MagNA Pure LV DNA isolation kits were used. The saliva samples collected after the second season of the trial in the Gambia were extracted using a Maxwell 16 instrument (Promega, USA) and Maxwell 16 DNA purification kits. Concentration measurements were done using a NanoDrop device (Thermo Fisher, USA) (only DNA from full blood in EDTA) and a Qubit fluorometer (Thermo Fisher, USA) with the Qubit HS (high-sensitivity) kit, which is specific for double-stranded DNA (dsDNA).

### Gametocyte detection.

Quantitative nucleic acid sequence-based amplification (QT-NASBA) was performed as described previously by Schneider et al. (34), and quantitative reverse transcription-quantitative PCR (qRT-PCR) was performed as described previously by Wampfler et al. (35). Briefly, total NA was used for amplification of the P. falciparum mature gametocyte marker Pfs25 mRNA for the estimation of mature gametocyte density in samples from the clinical trials. Gametocyte densities were assigned based on plate-specific gametocyte dilution series, which were diluted in whole blood before extraction of total NA, as with the samples from the clinical trials. For samples from trial participants, estimated gametocyte densities below 0.02 gametocytes per μl were considered to be negative.

### Ethical considerations.

Informed consent was obtained from all study participants. The studies received approval from the Ethics Committee of the Faculty of Medicine, Pharmacy, and Dentistry, University of Science, Techniques and Technologies of Bamako, and the Committee on Human Research at the University of California, San Francisco (studies in Mali); the Comité d’Ethique pour la Recherche en Santé, Ministère de la Santé du Burkina Faso, and the Comité Technique d’Examen des Demandes d’Autorisation d’Essais Cliniques, Ministère de la Santé du Burkina Faso (studies in Burkina Faso); the Gambia Government/MRC Joint Ethics Committee (studies in the Gambia); the Makerere University School of Medicine research ethics committee and the Uganda National Council of Science and Technology (study in Uganda); the Kenya Medical Research Institute Ethics Review Committee (study in Kenya); and the Interventions Research Ethics Committee of the London School of Hygiene and Tropical Medicine (all studies).

### CYP2D6 metabolizer status.

Samples with sufficient quantities of DNA (50 ng/μl without or 2.5 ng/μl with the manufacturer-provided preamplification kit) were genotyped for *CYP2D6* *2, *3, *4, *6, *7, *8, *9, *10, *11, *15, *17, *18, *19, *20, *29, *40, and *41 alleles using OpenArray technology on a QuantStudio 12K Flex RT-PCR system (Life Technologies, Carlsbad, CA, USA). The *CYP2D6* copy number was determined with at least one TaqMan copy number assay targeting intron 2 (Hs04083572_cn), intron 6 (Hs04502394_cn), and/or exon 9 (Hs00010001_cn), depending on the available sample quality and volume. CYP2D6 metabolizer status was inferred from the genotypes using the activity score (AS) (36). An AS of 0.0 indicates a poor metabolizer (PM), an AS of 0.5 to 1.0 indicates an intermediate metabolizer (IM), an AS of 1.5 to 2.0 indicates an extensive metabolizer (EM), and an AS of >2.0 indicates an ultrarapid metabolizer (UM) (37). For the analyses, we compared PM/IM versus EM/UM.

### Statistical analysis.

As a single measure of PQ efficacy, we used the presence of gametocytes on either day 7 or day 10. The effect on Hb was quantified in two ways: the day 7 Hb concentration (from studies with day 7 measurements) and the minimum observed Hb concentration (from all studies; up to day 28 after initiation of treatment). Because different trials used different PQ doses, the PQ dose was categorized as no PQ (control arms), 0.25 mg/kg PQ (0.10 to 0.25 mg/kg), 0.5 mg/kg PQ (0.4 to 0.5 mg/kg), or 0.75 mg/kg PQ. Anemia was defined based on criteria of the World Health Organization (38): moderate anemia was defined as an Hb concentration of <11 g/dl for adults or <10g/dl for children <5 years of age, and severe anemia was defined as an Hb concentration of <8 g/dl for adults or <7g/dl for children <5 years of age. Logistic and linear regression models were used to analyze the effect of CYP2D6 status on gametocyte prevalence, anemia, and Hb concentration. Models controlled for PQ dose, study, baseline gametocyte and asexual parasite densities (in efficacy analyses), and baseline Hb concentration (in safety analyses).

## Supplementary Material

Supplemental file 1

Supplemental file 2
